# An assessment of vulnerability to HIV infection of boatmen in Teknaf, Bangladesh

**DOI:** 10.1186/1752-1505-2-5

**Published:** 2008-03-14

**Authors:** Rukhsana Gazi, Alec Mercer, Tanyaporn Wansom, Humayun Kabir, Nirod Chandra Saha, Tasnim Azim

**Affiliations:** 1 Health Systems and Infectious Diseases Division, ICDDR, B, Mohakhali Commercial Area, Dhaka 1212, Bangladesh; 2 Laboratory Sciences Division, ICDDR, B, Mohakhali Commercial Area, Dhaka 1212, Bangladesh; 3 University of Michigan Medical School, Ann Arbor, Michigan, USA

## Abstract

**Background:**

Mobile population groups are at high risk for contracting HIV infection. Many factors contribute to this risk including high prevalence of risky behavior and increased risk of violence due to conflict and war. The Naf River serves as the primary border crossing point between Teknaf, Bangladesh and Mynamar [Burma] for both official and unofficial travel of people and goods. Little is known about the risk behavior of boatmen who travel back and forth between Teknaf and Myanmar. However, we hypothesize that boatmen may act as a bridging population for HIV/AIDS between the high-prevalence country of Myanmar and the low-prevalence country of Bangladesh.

**Methods:**

Methods included initial rapport building with community members, mapping of boatmen communities, and in-depth qualitative interviews with key informants and members from other vulnerable groups such as spouses of boatmen, commercial female sex workers, and injecting drug users. Information from the first three stages was used to create a cross-sectional survey that was administered to 433 boatmen.

**Results:**

Over 40% of the boatmen had visited Myanmar during the course of their work. 17% of these boatmen had sex with CSW while abroad. There was a significant correlation found between the number of nights spent in Myanmar and sex with commercial sex workers.

In the past year, 19% of all boatmen surveyed had sex with another man. 14% of boatmen had participated in group sex, with groups ranging in size from three to fourteen people. Condom use was rare {0 to 4.7% during the last month}, irrespective of types of sex partners. Regression analysis showed that boatmen who were 25 years and older were statistically less likely to have sexual intercourse with non- marital female partners in the last year compared to the boatmen aged less than 25 years. Similarly deep-sea fishing boatmen and non-fishing boatmen were statistically less likely to have sexual intercourse with non- marital female partners in the last year compared to the day long fishing boatmen adjusting for all other variables. Boatmen's knowledge regarding HIV transmission and personal risk perception for contracting HIV was low.

**Conclusion:**

Boatmen in Teknaf are an integral part of a high-risk sexual behaviour network between Myanmar and Bangladesh. They are at risk of obtaining HIV infection due to cross border mobility and unsafe sexual practices. There is an urgent need for designing interventions targeting boatmen in Teknaf to combat an impending epidemic of HIV among this group. They could be included in the serological surveillance as a vulnerable group. Interventions need to address issues on both sides of the border, other vulnerable groups, and refugees. Strong political will and cross border collaboration is mandatory for such interventions.

## Introduction

Mobile populations such as truckers and migrant workers are at risk for contracting HIV infection [1-3]. Many different factors, including time spent away from home, may contribute to risky behaviour among mobile occupational groups. For example, a study among truck drivers in South India found that an increased duration of trips was a significant risk factor for HIV infection [1]. However, a study in Brazil reported a high prevalence of risk behaviour among short route truck drivers, suggesting that other factors were involved in increasing vulnerability to HIV infection [4]. Sailors and fishermen constitute mobile population groups spending both long and short periods away from home.

Risky sexual behaviour and high prevalence of HIV [3.2% to 16.1%] has previously been documented among fishermen groups [5-9]. Often fishermen communities have higher prevalence of HIV compared to the general population [6,10-13]. In Malaysia, HIV prevalence among fishermen is 7.8%, while in the general population, is less than 2% [10]. A cross-sectional study of migrant fishermen in Thailand conducted in 2000 found a 15% prevalence of HIV/AIDS, while a 2004 cross-sectional study among fishermen in Sihanoukville, Cambodia documented an HIV prevalence of 16.1%, more than double the HIV prevalence estimated for the general population [6,11]. HIV/AIDS is identified as the leading cause of death among adults aged between 15 and 50 in Lakeshore areas in Uganda [12]. In Kagera region, Tanzania, fishermen were found to be five times more likely to die of AIDS-related illnesses than farmers [13]. Despite the high vulnerability to HIV infection of fisher community when compared to the general population, awareness regarding the causes of HIV infection is reported to be low [14]. A study done among fishermen in the Gulf of Thailand and the Andaman Sea found 13% self-treatment of last STD [Sexually Transmitted Disease] [15].

A comprehensive review of fishermen communities by Alison and Seeley suggested that their vulnerability to HIV stems from complex, interacting causes that may include the time fishermen spend away from home, their high mobility, their access to daily cash income in an overall context of poverty, their background characteristics, the ready availability of commercial sex in fishing ports and risk taking behaviors [8]. Factors such as young age, unmarried status, multiple sex partners including commercial sex partners, duration of stay in the port areas, lack of condom use, and prevalence of sexual violence have been found to be associated with increased vulnerability of fishing communities to HIV infection [5,9,11]. Since some studies in fishing communities have reported high HIV prevalance in women [5,10], it is assumed that heterosexual transmission prevails as a source of infection in such communities. However, other risk behaviors, such as injecting drug use, male to male sex, and tattooing have been found to play important roles in fueling the epidemic in many parts of the world among fishermen groups [16,17].

Bangladesh is surrounded by a high HIV prevalence neighboring country at southern part, Myanmar [18]. Teknaf is a small town in the Chittagong Division at the southern tip of Bangladesh, separated from Myanmar on the eastern side by the river Naf. The Naf River serves as a primary crossing point for people traveling back and forth between Bangladesh and Myanmar. Teknaf, Bangladesh, a burgeoning tourist spot, has about 23,000 inhabitants and is situated along the bank of the Naf River, with the Arakan state of Myanmar on the other side. Although Bangladesh currently has a low prevalence of HIV/AIDS [19], there is a potential for the spread of HIV across the border areas of Teknaf because of high cross border mobility. Dockworkers have reported high-risk behaviour such as unsafe sex; however, information on the fishermen and boatmen communities in Teknaf is scarce [20].

This border area is unique for many reasons, including the history of the tens of thousands of refugees that are currently living in squalid conditions on the Bangladeshi side. These refugees are comprised of large numbers of a Muslim minority, also known as Rohingyas that had previously lived in Myanmar's Arakan state for generations. After the Myanmar military came into power the Rohingyas were required to leave their homes and relocate to areas away from the border. More than a quarter million people fled to Bangladesh after 1962. About 22,000 of these refugees live in one of two camps managed by the UN Refugee Agency (UNHCR), but many others are undocumented and attempt to make a living in professions such as fishing, boating, smuggling, and sex work. Bangladesh refuses to recognize both official and unofficial refugees as citizens, rendering the vast majority of the permanent population in this border area stateless [21]. In addition to the high numbers of refugees in this area, there has also been an increase in the numbers of seasonal and permanent people that move back and forth between Bangladesh and Myanmar. The border remains very porous despite efforts at regulation on both sides. Since 1979, the Myanmar port has officially been open for bilateral communication between Bangladesh and Myanmar. Bangladeshi people can get a single entry visa for 1 to 5 days from the local office to visit Myanmar [21].

The rise of the commercial sex industry has mirrored the growth of Teknaf as a tourist spot and border crossing. Of ten hotels in Teknaf, eight were identified as linked with the sex trade. Residence-based sex workers are located in and around Teknaf. In addition, refugee camps are known to be common places for commercial sex. Boatmen and fishermen are anecdotally reported to be common customers of all types of sex workers in Teknaf. In the proposed study, we aim to assess whether boatmen in Teknaf are vulnerable to HIV infection by quantifying the prevalence of risky behaviour in a border area close to a high prevalence country. By placing such information in a broader context, we also hope to characterize linkages with other high-risk communities that could fuel the spread of an HIV epidemic in this area. Such information is relevant for developing and designing intervention programmes to prevent such an epidemic, and create a basis for a decision about the need for serological testing. Most importantly, it highlights the need for governments to come together to address root causes of health issues on both sides of the border [21].

## Methods

The study was conducted from April 2005 to December 2005. Preliminary qualitative information was collected through census of the fishing spots and in-depth interviews of both key informants as well as boatmen themselves. This information helped guide the development of the questionnaire for the quantitative survey. Survey data was collected through structured interviews.

### Preliminary data collection

As a part of rapport building, initial meetings were conducted with relevant people of the community. These meetings allowed the study team to identify local vulnerable groups and key informants who were linked with boatmen. Follow-up meetings were conducted with selected key informants during the process of data collection.

A mapping exercise was conducted to construct a census of boats and boatmen in Teknaf. Information was collected through observations made by trained interviewers. Preliminary information on number, location, and types of 'ghats' (landing areas/platforms for boats) was obtained from key informants. Field workers visited each 'ghat', counted boats, boatmen, and made observation for four to five hours at each ghat to identify the types of boatmen according to their activities. Each 'ghat' was observed twice. Mapping information was crosschecked with prior information obtained from key informants.

The mapping exercise identified three types of boatmen based on their activities: non-fishing boatmen, day-long fishing boatmen, and deep sea fishing boatmen. Thirty-three non-fishing boatmen from fifteen boats were identified at two ports in Teknaf. These boatmen originate in Teknaf and travel to Myanmar transporting either passengers or goods. Day long fishing boatmen were defined as non-migrant boatmen who fish in the local areas but do not go to the deep sea for fishing. Deep sea fishing boatmen are those who spend 7–10 days in the deep sea on fishing expeditions.

In-depth interviews were conducted with selected key informants; elderly boatmen, boat owners, NGO workers, male and female pimps of sex workers, journalists, hotel managers, and border traders. Information provided by different key informants was compared to identify contradictions and points of consistency regarding information provide.

Another series of in-depth interviews were conducted in order to further elaborate the risk behaviour linkages of Teknaf boatmen and obtain more information about their lifestyle and day-to-day experiences. These interviews were conducted with members of the following groups: boatmen (n = 17), spouses of boatmen (n = 11), female commercial sex worker (CSW) from different venues, including street, hotel, and home-based workers (n = 31), injecting drug users (n = 11), and transport workers (n = 5). In-depth interviews were conducted using flexible guidelines, which were developed after obtaining preliminary information from mapping. The recorded interviews were transcribed. Content analysis was done manually with the transcripts. Similar themes and sub themes were identified and categories. To describe linkages between patterns, themes, and experiences, we developed a data matrix with different cells to reflect the relationships identified within the data.

### Cross-sectional survey

For the sample size estimation, we assumed 3% condom use rate, as last time condom use rate during sex with CSW by transport worker was reported to be 3% [19], with precision of +/-2%, and a level of confidence of 95%. Based on these parameters, the sample size required was 279. Assuming a non-response percentage of 5%, 294 boatmen would need to be contacted. Values of other key indicators, such as sexual intercourse with CSW, are not known. However, a sample of 279 interviews would provide adequate precision with an estimated prevalence of sex with a commercial sex worker of 50% (+/-5.8%) and greater precision on lower prevalence estimates. As mapping exercises identified 33 non-fishing boatmen and about 900 deep sea fishing boatmen, we attempted to enroll all non-fishing boatmen and one third of the deep sea fishing boatmen. There were two ports for deep-sea fishing and each deep sea fishing boats consisted of three types of boatmen such as pilot, enginemen, and other crewmembers. There were 10 to 12 boatmen in each boat. We targeted 25 boats from each port and recruited 6 boatmen from each boat covering three types of crewmembers. We also enrolled 104 non-migrant local boatmen who were involved with the local fishing trade to have a comparison as we assumed that they have lower risk of HIV infection due to cross border mobility.

Data was collected by 8 trained interviewers having minimum a bachelor degree, who were previously involved in the national behavioral surveillance for HIV. In addition, fifteen days intensive training was given to the data collectors on vulnerable groups in the study site, risk behaviour for HIV, mode of transmission of HIV infection, and data collection techniques including data collection tools. The cross-sectional survey collected demographic information, looked at boatmen's mobility patterns, and assessed boatmen's knowledge about HIV transmission and their own perceptions of risk. The survey measured vulnerability through data regarding boatmen's sexual risk behavior, including number, gender, and types of sexual partners and condom usage with different types of partners. The survey questionnaire consisted of seven sections. The first section was on socio demographic profile of the boatmen. The second section inquired about knowledge of boatmen related to HIV infection particularly on modes transmission, risks, and prevention. The third section included questions regarding experience of symptoms of sexually transmitted infections and its care. In section 4, use of condom was investigated. Section 5 and 6 addressed the issues of sexual practices and sexual partners. The last section was on sexual violence.

Boatmen were defined as males aged 18 and over who had worked in the past six months as crewmembers in a boat based in Teknaf. Boats were defined as those used primarily for fishing for transport of passengers, or for trade of goods. Participation in the study was on a voluntary basis. All participants in the study received a simple explanation about the objectives of the study and oral consent was obtained prior to the in-depth qualitative interviews or the quantitative survey. Confidentiality was strictly maintained throughout the interview process. Permission to tape record interviews were obtained from the participants prior to the interview took place. During the few in-depth interviews where tape recording was not possible detailed notes were taken instead. All surveys were conducted anonymously. Prior to the implementation of the study, official approval was obtained from the Research Review Committee (RRC) and the Ethical Review Committee (ERC) of ICDDR, B. The ERC is an independent body house at ICDDR, B to look after the ethical aspects of research protocols of the Centre and comprised of experts mostly outside the Centre.

Data was entered using Epi-Info for Windows Version 3. Range and consistency checks were done to ensure accuracy. Data was analyzed using SPSS version 10. Both bi-variate and multivariate analysis were done. Descriptive analysis included preparation of frequency cross-tabulations, calculating means, medians and 95% confidence intervals on estimated proportions for categorical variables. In bi-variate analysis demographic characteristics of boatmen are presented and Student-t test was done to see the overall significance of different categories. Also, chi-square/fisher's exact test performed and crude odds ratios with 95% CI obtained to see the association between sexual intercourse with non-marital partners and other variables such as marital status, age, education, income, type of boatmen, activities of boatmen, knowledge of mode of transmission of HIV, and individual risk perception. In multivariate analysis logistic regression was done to assess the influence of co-factors such as marital status, age, education, income, type of boatmen, activities of boatmen, knowledge of mode of transmission of HIV, and individual risk perception on dependent variable that is intercourse with female non-marital partners in last 12 months. To show statistical significant associations between impendent and dependent variables adjusted odds with 95% CI were calculated and p-values were obtained using logistic regression model.

## Results

### Background characteristics of the boatmen

Surveys were completed by 433 boatmen. Table 1 shows the background characteristics of the boatmen by type (deep sea fishing, local fishing, and non-fishing boatmen). Overall, the mean age of the boatmen ranged between 26 to 28 years. Thirty nine percent of the boatmen were between the ages of 18–24. A vast majority of the boatmen (73%) had no formal education. In comparison to other groups, the non-fishing boatmen were more likely to be under age 24, unmarried, and earn very low incomes.

**Table 1:** Background characteristics of boatmen by groups

Variables	Deep Sea boatmen n = 300	Day long fishing boatmen n = 104	P- value	Non-fishing boatmen n = 29	Total
Age in years					
18 – 24	39.3	37.5		48.3	39.05
25–34	38.3	48.4		34.5	40.4
35+	22.3	14.4	0.120	17.2	20.1
Mean ± SD	28.1 ± 8.6	26.2 ± 5.9		26.1 ± 10.6	
Marital status					
Married	66.0	70.2		48.3	65.8
Single	34.0	29.8	0.433	51.7	34.2
Education in years					
No formal education	76.0	66.3		65.5	73.0
1–5 grade [primary]	19.0	30.8		31.0	22.6
6–9 grade [secondary]	5.0	2.9	0.037	3.4	4.4
Activities as boatmen					
Pilot	30.7	12.5		51.7	27.3
Helper	26.0	12.5		48.3	24.2
Fishermen	27.0	62.5		0	33.7
Engine man	16.3	12.5	0.000	0	14.8
Monthly income in Taka					
< US$ 44	13.3	22.1		58.6	18.5
US$ 44–59	39.3	61.5		34.5	62.8
US$ 59+	47.3	16.3	0.000	6.9	18.7
Mean ± SD	69 ± 34	52 ± 17		39 ± 24	

### Sexual behaviour of the boatmen with non-marital female sexual partners

Non-marital female sexual partners are defined as sex partners who are not paid for sex (both casual and regular partners) as well as CSW. Characteristics of the boatmen's sexual behavior with non-marital female sexual partners are shown in Table 2. The table divides boatmen into groups based on type as well as marital status. In the last twelve months, 36 to 60% of all boatmen had two or more non-marital female sex partners. Overall, 34.8% of married men reported sexual intercourse with a non-marital female partner in the last year. However, a higher proportion of unmarried boatmen compared to the married groups tended to have sex with non- marital female sexual partners, odds ratio (OR) 0.59, 95% confidence interval (CI) (0.38–0.89). Table 2 shows boatmen's sexual behaviour with non-marital female partners by different boatmen categories. Higher proportion of unmarried boatmen than married tended to have sexual intercourse in with any other non-marital partners in all groups during the last year. Of the non-marital female partners, 78 to 81% were sex workers (not shown in the table). Unmarried boatmen were more likely to have sexual intercourse with sex workers in last 12 months when compared to married boatmen. In the last month alone, 27% of all boatmen reported to having had sexual intercourse with sex workers.

**Table 2:** Boatmen's sexual behaviour with non-marital female partners

Variables	Deep sea fishing boatmen		Day long fishing boatmen		Non-fishing boatmen		Total
	
	Married n = 198	Single n = 102	Married n = 73	Single n = 31	Married n = 14	Single n = 15	n = 433
Proportion of boatmen who had sexual intercourse with non-marital female partners in last 12 months	48.5 [41.5–55.4]	60.8 [51.3–70.2]	67.1 [56.3–77.8]	80.6 [66.6–94.5]	42.9 [16.9–68.8]	33.3 [9.4–57.1]	57.3
	P = .043		P = .164		P = .594		

Proportion of boatmen who had sexual intercourse with non-marital female partners in last month	30.8 [24.3–37.2]	46.1 [36.4–55.7]	34.2 [23.3–45.0]	58.1 [40.7–75.4]	0	26.7	35.8
	P = .009		P = .023				

Proportion of boatmen who had sexual intercourse with CSW in last 12 months	38.9 [32.1–45.6]	45.1 [35.4–54.7]	57.5 [46.1–68.8]	70.0[53.9–86.1]	42.9 [16.9–68.8]	44.8 [19.6–69.9]	46.1
	P = .301		P = .231		P = .917		

Proportion of boatmen who had sexual intercourse with CSW in last month	24.2 [18.2–0.1]	32.4 [23.3–41.4]	30.1 [19.5–40.6]	38.7 [21.5–55.8]	0	26.7	27.5
	P = .392		P = .392				

Number of non-marital female partners in last 12 months							

1	12.1 [7.5–6.6]	8.8 [3.3–4.2]	6.8 [1.2–12.5]	6.4 [2.2–15.0]	7.1 [6.3–20.5]	20.0 [.2–40.2]	10.2
	P = .386		P = .940		P = .313		

2+	36.4 [29.6–43.1]	52.0 [42.3–61.6]	60.3 [49.0–71.5]	74.2 [58.7–89.6]	35.7 [10.6–60.7]	46.7 [21.4–71.9]	47.1
	P = .009		P = .175		.547		

Note: Confidence Interval [CI] is shown within parenthesis, corresponding p value is given below

In univariate analysis demographics of the boatmen were associated with a higher likelihood of having sex with a non-marital female partner in the last twelve months shown in Table 3. After adjusting for marital status, education, income, types of boatmen, knowledge of mode of transmission, and individual risk perception, boatmen who were 25 years and older were statistically less likely to have sexual intercourse with non- marital female partners in the last year compared to the boatmen aged less than 25 years. Similarly deep-sea fishing boatmen and non-fishing boatmen were statistically less likely to have sexual intercourse with non- marital female partners in the last year compared to the day long fishing boatmen adjusting for all other variables.

**Table 3:** Factor associated with boatmen's sexual intercourse with any non-marital female partners in last 12 months

Independent variables	Crude odds ratio (95%CI)	Adjusted odds ratio (95% CI)	P-value
Marital status			
Married†			
Single	1.68(1.12–2.55)	1.20(0.68–2.09)	0.528
Age group in years			
Age < 25 yrs †			
25+ yrs	0.48(0.32–0.72)	0.47(0.27–0.81)*	0.007
Educational attainments			
Had formal schooling †			
Had no formal schooling	1.63(1.05–2.54)	1.48(0.92–2.38)	0.106
Income			
< 3000 Taka †			
3000+ Taka	0.55(0.33–0.92)	0.62(0.34–1.12)	0.116
Type of boatmen			
Local fishing †			
Deep sea fishing	0.53(0.34–0.82)	0.35(0.20–0.60)*	0.000
Non-fishing	0.91(0.43–1.95)	0.25(0.94–0.69)*	0.007
Types of activities of boatmen			
Pilot †			
Helper	1.22(0.78–1.97)	0.93(0.51–1.71)	0.825
Fisherman	0.93(0.62–1.40)	0.55(0.30–0.99)	0.050
Engineman	1.29(0.75–2.23)	1.03(0.52–2.04)	0.928
Knowledge on mode of transmission of HIV			
Knew HIV could be transmitted through sexual intercourse †			
Did not know HIV could be transmitted through sexual intercourse	1.19(0.59–2.93)	1.15(0.52–2.52)	0.735
Individual risk perception			
Perceived risk of being infected with HIV †			
Perceived no risk for self	0.86(0.56–1.33)	0.66(0.40–1.09)	0.106

* Statistically significant

† Reference category

Rates of condom usage with non-marital female partners are shown in Table 4. Overall, small proportions of boatmen reported using condom during sexual intercourse during sex with non-marital partners. The proportion of daylong fishermen who used condoms during sex with non-marital female sexual partners in the last 12 months was lowest among all groups. Only 3.3% of all boatmen reported always using condoms during sex with commercial female sex workers in the last month.

**Table 4:** Condom use during sex with female non-marital partners

Variables	Deep Sea fishing boatmen	Day Long fishing boatmen	Non-fishing boatmen	Total number
	n = 164	N = 74	n = 22	n = 260
Proportion of boatmen who used condoms during sex with non-marital female partners in last 12 months (denominator is who had sex with non-marital partners in last 12 months)	11.0	4.1	18.2	9.6
Proportion who used condoms during sex with non-marital female partners in last month (denominator is who had sex with non-marital partners in last month)	n = 108	N = 43	n = 4	n = 155
Never	63.0	9.3	75.0	71.6
Sometimes	32.4	2.3	25.0	23.9
Always	4.6	4.7	0	4.5
Proportion who used condoms during sex with CSW in last month (denominator is who had sex with commercial female sex workers in last month)	n = 82	n = 35	n = 4	n = 121
Never	64.6	94.3	75.0	73.6
Sometimes	31.7	2.9	25.0	23.1
Always	3.7	2.9	0	3.3
Proportion who used condoms during sex with CSW at last visit to sex worker (denominator is who had sex with CSW in last 12 months)	n = 127	n = 64	n = 19	n = 210
	11.0	3.1	15.8	9.0

### Sexual behaviour of boatmen with male and transgender sexual partners

Characteristics of boatmen's sexual behavior with men and transgender sexual partners is shown in Table 5. Nineteen percent of boatmen reported that they had sex with other men in the last year. Approximately eight percent had sex with other men in the last month. About 5% of boatmen reported to having sex with a transgender person in the last year. Condom use was almost nil during sex with male and transgender sexual partners.

**Table 5:** Boatmen's sexual practices with men and transgendered partners

Boatmen's sexual practices with men and transgendered partners	Deep Sea fishing boatmen	Day long fishing boatmen	Non-fishing boatmen	Total
	n = 300	n = 104	n = 29	n = 433
Proportion who had sexual intercourse with men in last 12 months	20.0	21.2	10.3	19.6
Proportion who had sexual intercourse with men in last month	9.0	6.7	3.4	8.1
Proportion who had sexual intercourse with male sex workers in last 12 months	6.7	4.8	0	5.8
Proportion who had sexual intercourse with male sex workers in last month	4.0	1.0	0	3.0
Proportion who had sexual intercourse with transgender in last 12 months	4.7	2.9	0.3	4.6
Proportion who had sexual intercourse with transgender in last month	1.3	0	3.4	1.2

About fourteen percent of boatmen had participated in group sex during their lifetime. Group sex was defined as penetrative sex in a group session where there were at least two other partners. Boatmen reported to having group sex with groups ranging from three to thirteen partners.

### Reported symptoms relating to STIs

About 33% of the boatmen reported having STI symptoms in the last year. Reported signs and symptoms included penile discharge, dysuria (pain during urination), genital lesions, swelling in the groin area, and anal discharge. Of the boatmen who had STI problems in the last year, 16% did not seek any treatment. Boatmen who sought treatment commonly visited a pharmacist or a government facility.

### Knowledge of regarding HIV/AIDS

Only 30% of the boatmen had ever heard about HIV/AIDS. Of the boatmen who had heard about HIV/AIDS, they reported that their most common source of information about the disease was friends and relatives, followed by radio or TV.

Approximately 31% of boatmen knew that HIV/AIDS could be transmitted by sexual intercourse and 22% thought that sharing injecting equipment could be a mode of transmission for HIV/AIDS. Twenty-seven percent of boatmen knew that using condoms during sexual intercourse could prevent transmission of HIV/AIDS.

Only 14% of boatmen reported perceiving themselves to be at risk for contracting HIV at the time of interview. These boatmen identified risk to be associated with the following factors: having sex with CSW, having multiple sex partners, sharing needles or syringes, using condoms inconsistently or not at all, and sharing food with others Boatmen who did not think they were at risk for contracting HIV infection cited the following reasons for being protected against the virus; they washed their genitals with soap, water, or urine after sex, they selected partners who appeared healthy, they sometimes or always used condoms during sex, they did not share injection instruments, and they did not have sex with CSW. A few boatmen mentioned that they did not see others suffering from HIV so they did not think they themselves were at risk. A few boatmen also wore amulets, which they thought protected them from risk.

### Violence within the boatmen community

About 31% of the boatmen reported being beaten while working as a boatman. Five percent of boatmen reported being raped, with non-fishing boatmen comprising the majority of these cases. Non-fishing boatmen reported that forced sexual intercourse was commonly perpetuated by coastal guards, law enforcement agents, and sea pirates.

### Mobility of boatmen between Bangladesh and Myanmar

Overall 42% of the boatmen had been to Myanmar, with a mean number of 117 visits there. In the last three months, 100% of the non- fishing migrant boatmen, 35% of the deep-sea boatmen, and 31% of the daylong fishing boatmen had been to Myanmar. Of the boatmen who had visited Myanmar in the last three months, 39% reported to have sex with sex workers, and 4.2% had sex with other non-paying sexual partners. The mean duration of stay in Myanmar was three nights. There was a significant correlation observed between the numbers of nights stayed in Myanmar and sexual intercourse with sex workers while in Myanmar (statistically significant at .01 level). The most common place for overnight stays in Myanmar were boarding houses, which are not linked with the hotels of Teknaf.

Three percent of the boatmen who had previously been to Myanmar also reported traveling to other countries, including India, Pakistan, and Thailand.

## Qualitative results

### Sexual beliefs and practices of boatmen

In-depth interviews were conducted with sixteen boatmen to explore risk behaviour linkages, their lifestyles, day-to-day experiences, and risk perceptions. Five interviews were done with each of the deep sea and local boatmen and six interviews were done with daylong boatmen. Having multiple female sexual partners is perceived by most of the boatmen as symbolic of "strong masculinity." One commonly heard expression in interviews was, "*MoroderJibon"*, which states that men's lives are for enjoyment with girls..."Many boatmen stated "We have to face very dreadful conditions out in the sea, where our work is a matter of life and death. Because of this, we need to rejuvenate our energy." Sex with multiple sexual partners and drug use were seen as a means for 'rejuvenating energy'. Substances that were commonly abused included local wine, ganja (cannabis), heroin, and sleeping pills.

Boatmen believed that practicing sex in the deep sea was forbidden because they viewed the sea as pure and *"roti -ruji*", or a means for earning their livelihood. They believed that having sex in a boat that was out at sea would cause them to be cursed by the sea. This curse would extend to all aspects of his life and cause him extreme suffering. This belief caused many boatmen to engage in sexual activities exclusively around port areas. Both unmarried and married boatmen reported that they had both paid and unpaid sex partners in Teknaf and Myanmar. Premarital sex was found to be common in the boatmen community. CSW stated that many of their clients were boatmen. Both spouses of boatmen and CSW confirmed that condom (known as *fathna *in the local term) use was extremely rare. CSW were offered extra money to perform anal, oral, or group sex.

Almost all boatmen and some of the spouses experienced STI related symptoms in their life. For such problems mostly they sought treatments from *'kabiraj' *homeopathy practitioners or pharmacists. They do not prefer to consult health professionals because of two main reasons; one is lack of privacy/confidentiality to see those, another is they do not perceive such problems as serious health problems.

Although few boatmen heard about 'AIDS' but could not related it with route of transmission. Overall self-risk perception was low because most boatmen view HIV/AIDS as sort of a remote problem for them. One expression was "We don't see anybody known to us suffering from this disease, so why to worry?"

Only three boatmen reported any past history of injecting drug use. However, interviews with injecting drug users in Teknaf revealed that they occasionally shared injecting equipment with boatmen.

### Sexual linkages

In-depth interviews with other vulnerable groups including commercial female sex workers, injecting drug users, and transport workers revealed that boatmen are part of a risk behaviour network that begins locally in Teknaf and extends to Myanmar. Boatmen regularly interact with spouses, non-paying female sex partners, and commercial sex partners. The female CSW have other sexual partners such as transport workers and injecting drug users, many of whom have spouses and non-paying partners. Injection Drug Users (IUD) often share needle and syringes with their boatmen friends both in Teknaf and in Myanmar. Few local transport workers interviewed reported to work previously as boatmen and they still have linkages with boatmen community in Teknaf. Tourists from different parts of Bangladesh also stay in hotels in Teknaf where CSW are based. This broadens the network considerably.

As an extremely mobile group, boatmen make frequent visits to Myanmar both legally and illegally and often have sex with sex workers while in Myanmar, who they consider more attractive. Sex workers originally from Myanmar also move back and forth between Teknaf and Myanmar. They state that many women from Myanmar come to Teknaf seeking better job opportunities, higher wages, and prospects of marriage. Women from Myanmar often have unregistered marriages with men in Bangladesh. These women often get divorced or deceived by men and many of them are eventually compelled to join the sex trade.

The key informants confirmed that a complex web of social and sexual linkages bound boatmen between Teknaf and Myanmar and revealed the existence of many cross- border families between the two countries. They stated that boatmen who are originally from Myanmar (but are now citizens of Bangladesh) are preferred by employers because they have easy access to Myanmar. Officially, movement of Bangladeshi boatmen and traders with formal visas are restricted to "Modu ghat," or the landing platforms for boats, in Myanmar. However, this is not enforced and many stay overnight with relatives and friends.

### Limitation of the study

The study used a convenience sample, so there was a chance of having selection bias. However, as the boatmen are highly mobile, it was challenging to get access to the boatmen. Randomization of respondents was not possible.

## Discussion

Boatmen in Teknaf may act as a bridging population to fuel a future HIV epidemic in the Bangladesh/Myanmar border area. The present study is one of the first to describe and the vulnerability of boatmen in Teknaf to HIV by quantifying the prevalence of risky sexual behaviour and contextualizing it in a cross-border context. Many of the boatmen reported having multiple sexual partners, including paid and non-paid partners, both in Teknaf and Myanmar. Condom usage was found to be extremely rare during sex with all types of partners.

Boatmen in Teknaf are at high risk of contracting HIV infection through sexual linkages that connect Bangladesh and Myanmar, a country experiencing a generalized HIV epidemic. There is a great potential for boatmen infected with the virus to spread HIV to their spouses and other sexual partners (both male and female) in their communities. Local sex workers who cater to the needs of clients including boatmen are at even higher risk for contracting HIV as they attempt to strike a balance between making a living and protecting their health. Sex workers in Teknaf are often undocumented migrants from Myanmar, and are preferred in the local sex trade due to their attractive features. These women are extremely marginalized as a group and have the least power to negotiate for condom use. A study done in Hong Kong indicated that female migrant workers are often at risk of violence, discriminated against, and do not have adequate knowledge regarding HIV/AIDS [22]. The National Surveillance for HIV in Bangladesh also found that a large proportion of transport workers reported having both commercial and non-commercial sex partners and condom use was very low [19]. Sex workers may be even more vulnerable to violence at the hands of their clients because clients often come to them in drug or alcohol induced states. This underscores the prevailing norms of 'strong masculinity' and perceived means of 'rejuvenating energy' that encourages men to have sex with multiple partners and use alcohol and drugs, which increases both boatmen's and their partner's vulnerability to HIV/AIDS. In Bangladesh the highest HIV infection rate has always been recorded in injection Drug Users (IUD) that is at 4% [19]. Our study reported that some of clients of the sex workers were injection drug users. Also some of the IDU respondents mentioned that they often share injection equipment with boatmen, who were their friends. The present study demonstrates that boatmen in Teknaf are an integral part of a high-risk behaviour network between Myanmar and Bangladesh. In the national Surveillance for HIV, vulnerable groups such as transport workers, male and female sex workers, males who have sex with males and transgenders are included. Without having preliminary information on risk behaviour of a particular group, we could not recommend them to be included in the National surveillance. The present study has provided first hand information about such a vulnerable group and we encourage their inclusion into the National Serological Surveillance for HIV. It would provide stronger evidence whether they are at risk of acquiring HIV infection due to cross border mobility and sexual linkages.

Most boatmen in Teknaf lack a clear understanding of how HIV can be transmitted and how to protect themselves from contracting the disease. Despite high reporting of STI related symptoms by boatmen, they did not perceive that they were at risk for HIV. Many boatmen stated that HIV/AIDS was a remote problem to them because they did not see people suffering from the disease in their communities. Therefore, AIDS related education that addresses the needs of the boatmen in Teknaf must receive immediate attention from policymakers, researchers, and programmers. Interventions should be designed with a particular focus on mode of transmission of HIV infection. Such an intervention should also have components to promote safer sex practices that are community-driven. We recommend peer approaches to successfully reach boatmen communities within Teknaf. The peer boatmen might act as safe sex promoter through one to one communication or group discussions. They could also keep a store and distribute condom among other boatmen. One local NGO was identified working with injecting drug users and sex workers in Teknaf. But, no intervention was particularly targeted to boatmen in Teknaf. UNHCR can take a leadership role in establishment of local initiatives to address needs of such a vulnerable group as they are directly linked with local refugees community.

In conclusion, migration between the port towns of Teknaf, Bangladesh and Myanmar is prevalent. The geographical proximity of the two countries allows for a great deal of bilateral movement of people, goods, and illicit drugs. Furthermore, the border communities reflect the impact of widespread poverty, continuing conflict and persecution on both sides of the border, and a lack of health education and infrastructure. This study describes intricate risk behavior networks, which includes illicit drug use, multiple sex partners, unprotected sex, and violence. Regional approaches and collaborative efforts from those on both sides of the border are necessary for effective measures that will address the increased risk for a generalized HIV epidemic in this area.

## Authors' contributions

RG participated in the study design, coordinated the fieldwork, contributed in data analysis and drafted the manuscript. AM participated in design of the study. TW revised and modified the manuscript. HK implemented the study at field level and coordinated data collection. NcS participated in data management and data analysis. TA provided overall guidance to the study team. All authors approved the final manuscript.
